# Blockade of neuronal dopamine D2 receptor attenuates morphine tolerance in mice spinal cord

**DOI:** 10.1038/srep38746

**Published:** 2016-12-22

**Authors:** Wen-Ling Dai, Feng Xiong, Bing Yan, Zheng-Yu Cao, Wen-Tao Liu, Ji-Hua Liu, Bo-Yang Yu

**Affiliations:** 1 Jiangsu Key Laboratory of TCM Evaluation and Translational Research, China Pharmaceutical University, Nanjing, Jiangsu 211198, China; 2 Jiangsu Key Laboratory of Neurodegeneration, Department of Pharmacology, Nanjing Medical University, Jiangsu 211166, China; 3 State Key Laboratory of Natural Medicines, China Pharmaceutical University, Nanjing, Jiangsu 210009, China

## Abstract

Tolerance induced by morphine remains a major unresolved problem and significantly limits its clinical use. Recent evidences have indicated that dopamine D2 receptor (D2DR) is likely to be involved in morphine-induced antinociceptive tolerance. However, its exact effect and molecular mechanism remain unknown. In this study we examined the effect of D2DR on morphine antinociceptive tolerance in mice spinal cord. Chronic morphine treatment significantly increased levels of D2DR in mice spinal dorsal horn. And the immunoreactivity of D2DR was newly expressed in neurons rather than astrocytes or microglia both *in vivo* and *in vitro.* Blockade of D2DR with its antagonist (sulpiride and L-741,626, i.t.) attenuated morphine antinociceptive tolerance without affecting basal pain perception. Sulpiride (i.t.) also down-regulated the expression of phosphorylation of NR1, PKC, MAPKs and suppressed the activation of astrocytes and microglia induced by chronic morphine administration. Particularly, D2DR was found to interact with μ opioid receptor (MOR) in neurons, and chronic morphine treatment enhanced the MOR/D2DR interactions. Sulpiride (i.t.) could disrupt the MOR/D2DR interactions and attenuate morphine tolerance, indicating that neuronal D2DR in the spinal cord may be involved in morphine tolerance possibly by interacting with MOR. These results may present new opportunities for the treatment and management of morphine-induced antinociceptive tolerance which often observed in clinic.

Morphine is a highly efficacious agent against chronic severe pain. However, repeated morphine administration leads to antinociceptive tolerance which discontinues morphine therapy for chronic pain. The cellular and molecular mechanisms underlying morphine tolerance are still not fully understood. Morphine is known to exert analgesic effect mainly by activating MOR encoded by the MOR-1 gene. And in MOR-1 knockout mice, the analgesia and tolerance of morphine are absent^1^,^2^. These results suggested that morphine tolerance may be partly mediated by MOR and it has been reported that MOR desensitization^3^,^4^ contributes to the development of morphine tolerance. In addition, central sensitization also promotes the development and maintenance of morphine tolerance. Chronic morphine treatment can significantly increase the release of different neurotransmitters such as glutamate (Glu), which binds to the NMDA receptor to enhance the excitatory synaptic transmission^5^,^6^. It also activates the astrocytes and microglia^7^,^8^,^9^, leading to the release of the pro-inflammatory cytokines such as tumor necrosis factor-α (TNFα) and interleukin-1β (IL-1β)^10^,^11^,^12^ to promote the morphine tolerance.

D2DR, a dopamine receptor subtype which affects the locomotion, reward and abuse of opioids^13^,^14^,^15^,^16^ has been reported to be involved in morphine-induced nociception modulation in rats. Activation of D2DR by quinpirole in hypothalamic A11 cell group^17^, ventrolateral orbital cortex (VLO)^18^ and dorsal hippocampus (CA1)^19^ of rats exerts antinociceptive effect. Intraperitoneal injection of quinpirole enhances the antinociceptive effect of morphine^20^. However, Michael A. King and colleagues reported that the effect of opioid analegesia is potentiated in D2DR knock-out mice^21^. Therefore, the apparent analgesia effect and mechanism of D2DR were still not clear. And the effect and mechanism of D2DR on morphine tolerance has also not been elucidated.

The present study was performed to investigate the possible role of D2DR in morphine tolerance at spinal levels of mice. Our results indicated that chronic morphine treatment increased the neuronal D2DR expression and enhanced the MOR/D2DR interactions in the spinal cord. Intrathecal administration of D2DR antagonist sulpiride (1, 4 and 8 μg/10 μl), an antipsychotic drug used in clinic significantly attenuated chronic morphine induced tolerance and disrupted MOR/D2DR interactions in mice. The antinociception and morphine tolerance were assessed in Institute of Cancer Research (ICR) mice using tail-flick test. Cell signaling was assayed by western blot and immunofluorescence. The interactions between D2DR and MOR were evaluated by co-immunoprecipitation and immunofluorescence.

## Results

### Chronic morphine administration increases neuronal D2DR protein expression in mice spinal dorsal horn

**Figure 1:**
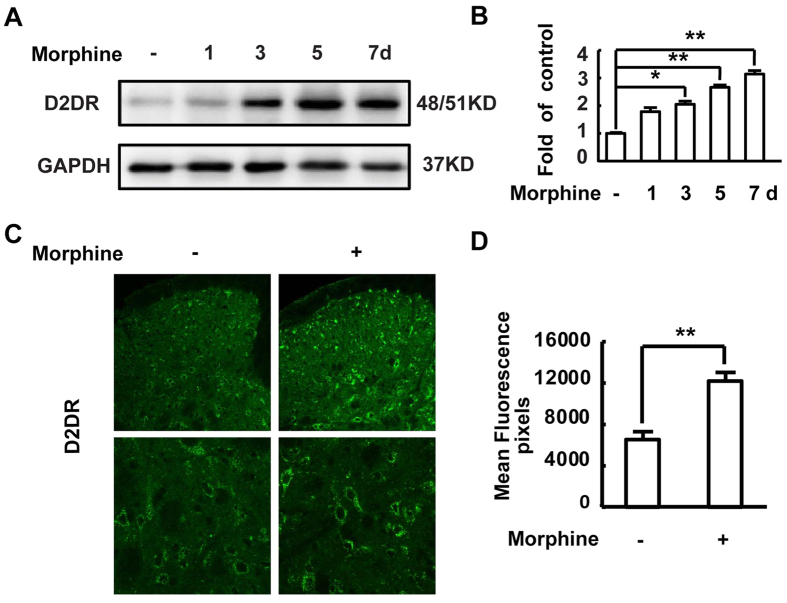
Chronic morphine treatment increases D2DR expression in mice spinal dorsal horn. (**A**,**B**) Chronic morphine treatment increased the spinal D2DR protein expression after morphine treatment for 3d, 5d and 7d (n = 4, *P < 0.05, **P < 0.01, compared with control group). Representative western blot bands and a data summary were shown. (**C**,**D**) Confocal images showed that the mean fluorescence pixels of D2DR were increased after chronic morphine treatment for 7 days. Quantification of D2DR immunofluorescence was represented as mean fluorescence pixels in the superficial dorsal horns. The spinal cord tissues were taken 30 min after morphine injection on day 1, 3, 5, 7. (*n* = 4, 8 images per animal; *P < 0.05, **P < 0.01 compared with morphine group).

Mice were treated daily with morphine (10 μg/10 μl, i.t.), and euthanized 30 min after daily intrathecal injection of morphine on days 1, 3, 5, and 7 to collect the lumbar cord for western blot analysis. The western blot data showed that D2DR protein expression increased time-dependently following chronic morphine treatment and was significantly upregulated after morphine treatment for 3 days (Fig. 1A and B). Consistent with this result, the immunofluorescence of D2DR protein expression showed the same tendency for D2DR protein elevation after intrathecal administration of morphine for 7 days (Fig. 1C and D).

**Figure 2:**
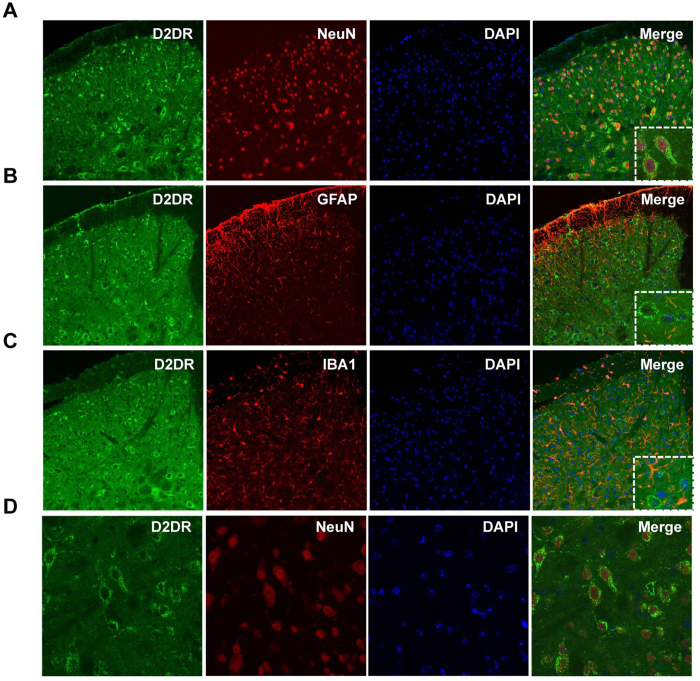
Distribution and co-localization of D2DR with NeuN, but not GFAP and IBA1 following chronic morphine exposure. (**A**,**D**) Confocal images showed that D2DR (green) co-localized with the neurons (red) in the spinal cord (20X magnification and 40X magnification). (**B**,**C**) It was difficult to see that D2DR co-localized with GFAP (red) or IBA1 (red) (20X magnification). All spinal cords were collected on day 7, 30 min after chronic morphine exposure (n = 4).

Double immunofluorescence labeling was performed to determine the localization of D2DR in mice spinal cord. Confocal images showed that D2DR immunoreactivity was co-localized with the mature neuronal marker NeuN, but not the astrocytic marker GFAP or the microglial marker IBA1 (Fig. 2). The results indicated that D2DR specifically expressed in mice spinal neurons.

### Pharmacological blockade of D2DR attenuates morphine antinociceptive tolerance and partly reverses established tolerance

**Figure 3:**
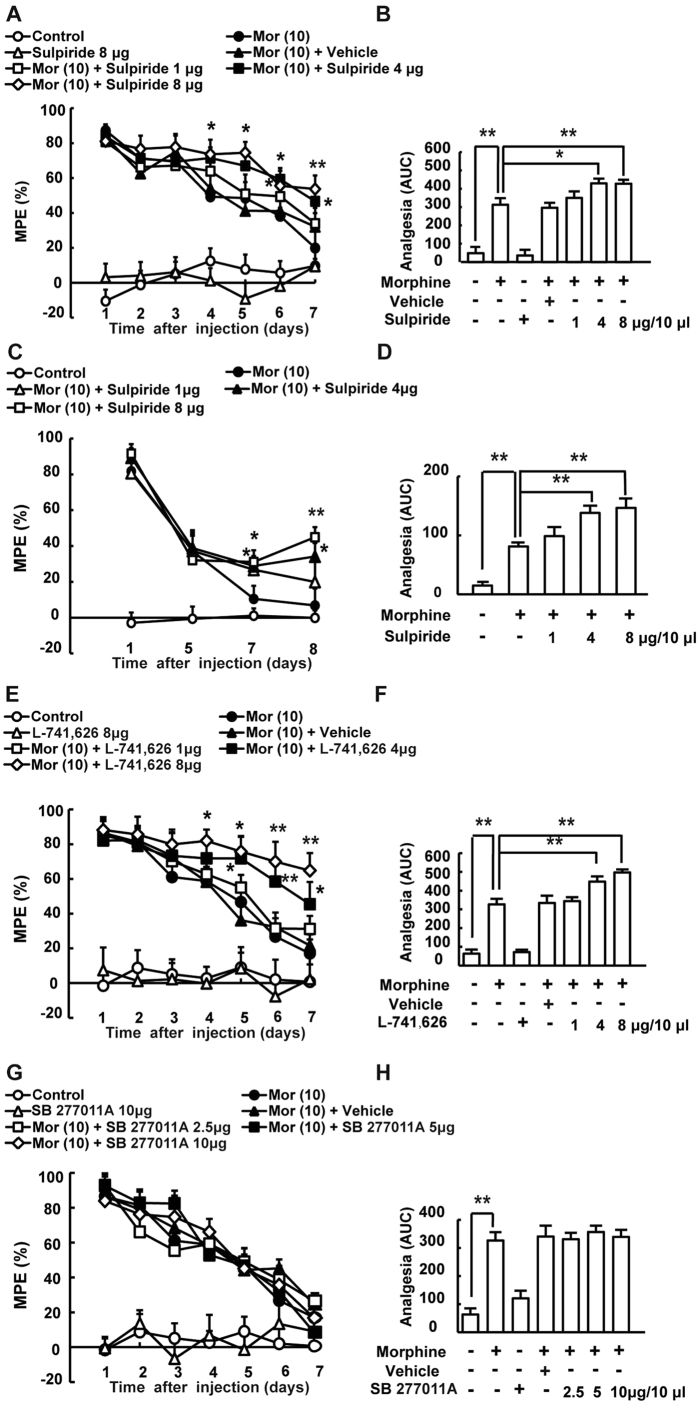
Daily intrathecal injection of D2DR antagonist attenuates morphine-induced antinociceptive tolerance. Mice were examined daily with tail-flick assay. Data were shown as percentage of maximal possible effect (% MPE). (**A**,**B**) D2DR antagonist sulpiride (1, 4 and 8 μg/10 μl, i.t.) attenuated the development of tolerance and exhibited no effects on the pain threshold in naive mice. (**C**,**D**) Sulpiride (1, 4 and 8 μg/10 μl, i.t.) partially reversed the established tolerance. (**E**,**F**) D2DR specific antagonist L-741,626 (1, 4 and 8 μg/10 μl, i.t.) also significantly attenuated morphine tolerance and exhibited no effects on the pain threshold in naive mice. (**G**,**H**) The specific receptor antagonist for dopamine D3 receptor SB 277011A (2.5, 5 and 10 μg/10 μl, i.t.) could not affect morphine tolerance in mice. (n = 12, *P < 0.05, **P < 0.01, compared with morphine group).

Chronic morphine administration increased D2DR expression in mice spinal cord. To assess the effect of D2DR blockade on morphine antinociceptive tolerance, tail-flick assay was used. The behavioral test showed that mice developed tolerance to the analgesic effect of morphine within 3 days after chronic intrathecal morphine treatment. Specific D2DR antagonist L-741,623, sulpiride (an antagonist for both D2 and D3 receptors) and D3 receptor antagonist SB 277011 were used here to determine their effect on morphine tolerance. Specific D2DR antagonist L-741,623 and sulpiride could attenuate morphine tolerance whereas D3 receptor antagonist SB 277011 had no effect on the development of morphine tolerance. The results indicated that antagonizing D2 receptor but not D3 receptor could attenuate morphine tolerance. Thus, sulpiride in this article mainly antagonized D2 receptor rather than D3 receptor to attenuate morphine tolerance. Since sulpiride has been used clinically and proved to be safe, we here used sulpiride as D2DR antagonist to further test its effect on morphine tolerance. The MPE (%) in morphine treated group on day 7 was 19.95 ± 4.25, whereas mice co-administrated with sulpiride (1, 4, 8 μg/10 μl, i.t.) had MPE (%) of 33.92 ± 8.96, 46.68 ± 6.45 and 53.69 ± 7.74 respectively. The analgesia was further represented by AUC units (Fig. 3A and B). L-741,626 could also attenuate morphine tolerance similar to sulpiride, the MPE (%) in morphine treated group on day 7 was 17.08 ± 8.02, and mice co-administrated with L-741,626 (1, 4, 8 μg/10 μl i.t.) had MPE (%) of 31.20 ± 7.48, 45.45 ± 8.59 and 64.81 ± 9.76 respectively (Fig. 3E and F). While the SB 277011 A (2.5, 5, 10 μg/10 μl) plus morphine group had MPE (%) of 17.08 ± 8.02, 26.52 ± 4.53, 8.61 ± 13.81 and 16.84 ± 10.74 respectively (Fig. 3G and H), suggesting that SB 277011 A could not attenuate morphine tolerance. Intrathecal delivery of sulpiride (8 μg/10 μl), L-741,626 (8 μg/10 μl), and SB 277011 (10 μg/10 μl) alone did not alter the pain threshold of mice, and even vehicle had no effect on the development of morphine tolerance.

In particular, intrathecal delivery of sulpiride (1, 4 and 8 μg/10 μl) was able to reverse established morphine tolerance. The behavioral test showed that mice developed morphine tolerance on day 5. Sulpiride (1, 4 or 8 μg/10 μl) was intrathecal delivery every 8 hours after morphine administration (10 μg/10 μl, i.t.) for 5 days. And from day 6, sulpiride (1, 4 or 8 μg/10 μl, i.t.) was administered 15 min before morphine challenge at 10 μg/10 μl. The results demonstrated that sulpiride (1, 4 or 8 μg/10 μl, i.t.) could reverse established tolerance. The MPE (%) in morphine treated group on day 8 was 6.77 ± 5.67, whereas mice co-administrated with sulpiride (1, 4, 8 μg/10 μl, i.t.) on day 8 had MPE (%) of 19.93 ± 9.50, 34.12 ± 7.57 and 44.88 ± 5.67 respectively. The analgesia was further represented by AUC units (Fig. 3C and D).

### Pharmacological blockade of D2DR decreases the phosphorylation of NR1, PKC, MAPKs and suppresses the activation of astrocytes and microglia induced by chronic morphine treatment

**Figure 4:**
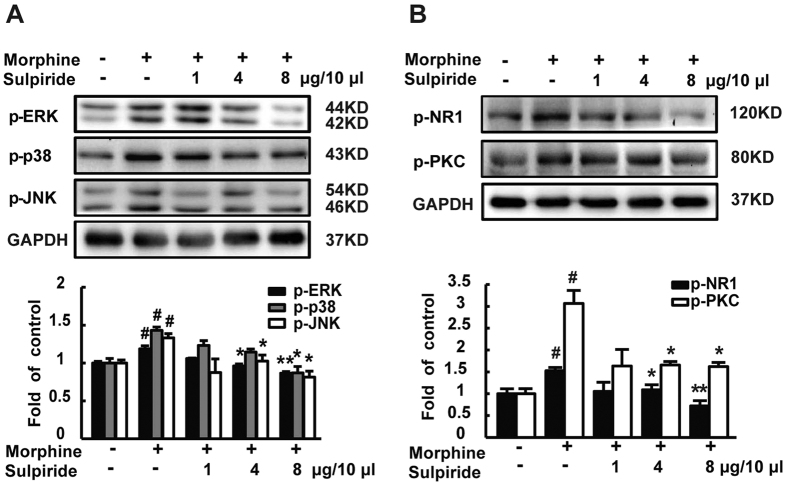
D2DR antagonist sulpiride attenuates morphine up-regulated phosphorylation of spinal NR1, PKC, ERK, p38 and JNK. (**A**) Sulpiride (1, 4 and 8 μg/10 μl, i.t.) decreased the up-regulated expression of p-ERK, p-p38 and p-JNK in the spinal cord following chronic morphine treatment. (**B**) Sulpiride (1, 4 and 8 μg/10 μl, i.t.) inhibited morphine induced up-regulated p-NR1, p-PKC expression in the spinal cord. Representative western blot bands and a data summary are shown. All spinal cords were collected on day 7, 30 min after chronic morphine exposure (n = 3, *P < 0.05, **P < 0.01, compared with morphine group).

NMDA receptors play a very important role in opiate-related neural plasticity^22^,^23^. Chronic morphine treatment has been reported to increase the levels of phosphorylated PKC^24^ and MAPKs^25^. Consistent with previous study, the levels of p-NR1, p-PKC, p-ERK, p-JNK and p-p38 were upregulated in mice spinal cord after chronic morphine treatment. Repeated co-application of sulpiride along with morphine significantly prevented or decreased morphine induced increase in p-NR1, p-PKC, p-ERK, p-JNK and p-p38 (Fig. 4A and B).

**Figure 5:**
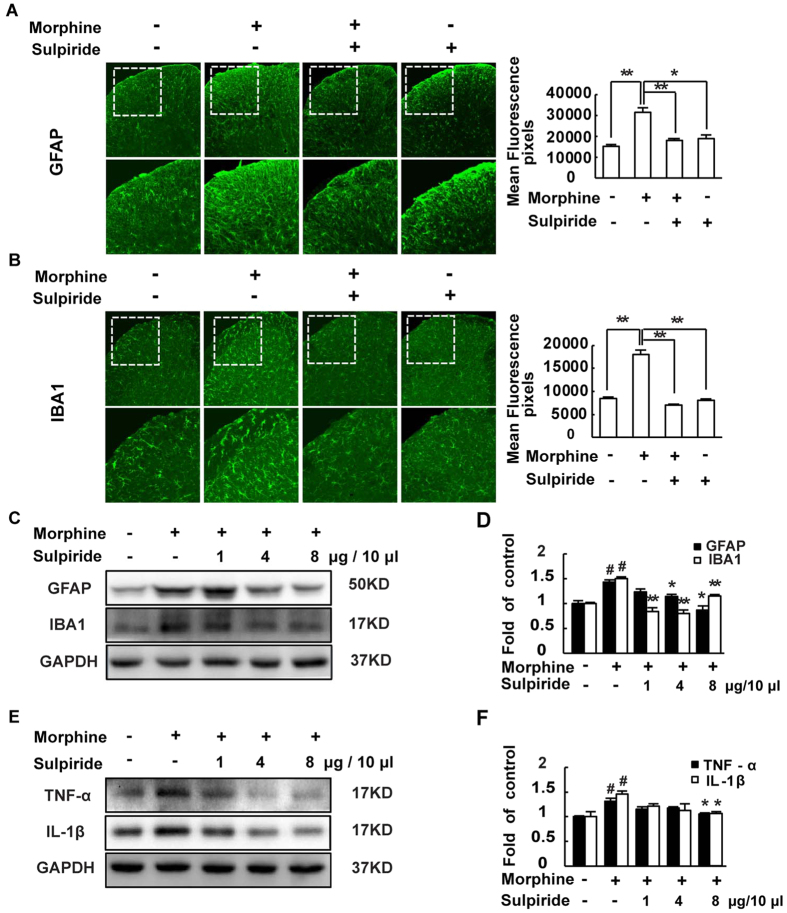
D2DR antagonist sulpiride suppresses morphine exposure induced activation of astrocytes and microglia in the spinal cord and decreases pro-inflammatory cytokines TNF-α and IL-1β. (**A**,**B**) Confocal images and immunofluorescence showed that chronic morphine treatment activated the astrocytes and microglia in the spinal dorsal horn. Sulpiride (8 μg/10 μl, i.t.) inhibited the activation of the glias in the spinal cord (10X and 20X magnification). Quantification of GFAP and IBA1 immunofluorescence were represented as mean fluorescence pixels in the superficial dorsal horns. (**C**,**D**) Western blot showed that sulpiride (1, 4 and 8 μg/10 μl, i.t.) decreased the up-regulated expression of GFAP and IBA1. (**E**,**F**) Western blot analysis data showed that the up-regulated expression of the pro-inflammatory cytokines TNF-α and IL-1β were decreased by sulpiride (1, 4 and 8 μg/10 μl, i.t.) (n = 3, *P < 0.05, **P < 0.01, compared with morphine group). Representative western blot bands and a data summary are shown. All spinal cords were collected on day 7, 30 min after chronic morphine exposure (n = 4, *P < 0.05, **P < 0.01, compared with morphine group).

Astrocytes and microglia are activated after chronic morphine treatment in mice spinal cord^7^,^8^,^9^, and the activated glias can increase the release of pro-inflammatory cytokines such as TNF-α and IL-1β^10^,^11^,^12^ to further promote the development of morphine tolerance. The western blot results showed that the levels of GFAP (an astrocytic marker), IBA1 (a microglial marker) (Fig. 5C and D) and TNF-α, IL-1β (Fig. 5E and F) were up-regulated after chronic morphine treatment. And the result of immunofluorescence showed the same tendency in the expression of GFAP and IBA1 (Fig. 5A and B). Sulpiride (1, 4 and 8 μg/10 μl, i.t.) significantly reduced the activation of the astrocytes and microglia and the release of TNF-α and IL-1β.

### Chronic morphine treatment increases the MOR/D2DR interactions in mice spinal cord

**Figure 6:**
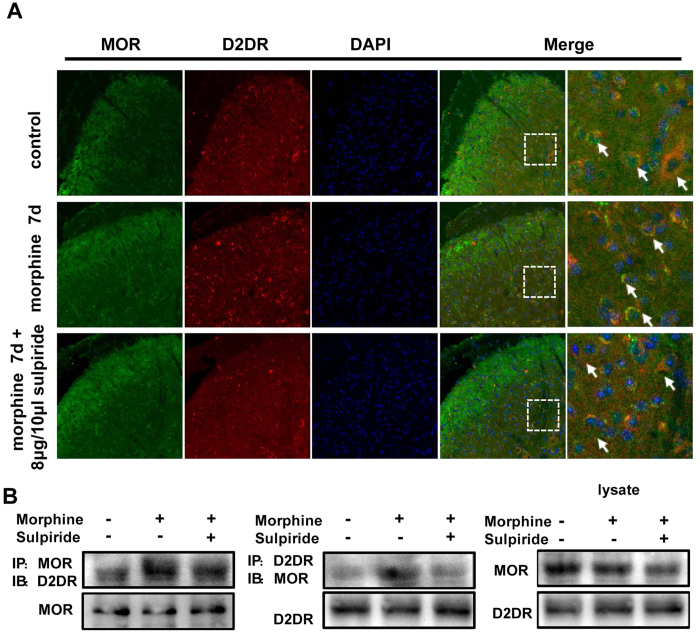
Chronic morphine treatment increases the MOR/D2DR interactions in the spinal cord dorsal horn and spinal D2DR inhibition with D2DR antagonist disrupts these interactions. (**A**) Double immunofluorescence staining showed that MOR (green) and D2DR (red) were co-localized in the mice spinal cord (20X magnification). Chronic morphine treatment increased the co-localization of MOR and D2DR in the spinal cord, and D2DR antagonist sulpiride (8 μg/10 μl, i.t.) reduced the increased co-expression of D2DR with MOR (n = 4). (**B**) Co-IP experiments showed that D2DR could interact with MOR, and the MOR/D2DR interactions were increased in the spinal dorsal horn after chronic morphine treatment for 7 days while D2DR antagonist sulpiride (8 μg/10 μl, i.t.) disrupted the interactions of the MOR/D2DR (n = 3).

Compelling evidences have accumulated that DOR can negatively modulate MOR activity by forming heterodimers with MOR in the spinal cord^26^,^27^. We wondered whether D2DR interacted with MOR to negatively regulate MOR activity and may be involved in μ-opioid antinociceptive tolerance. To elucidate whether D2DR and MOR were co-localized with each other in the spinal cord, double immunofluorescence staining was used. The result is that D2DR double-labeled with MOR in neuronal cell bodies, and this co-localization increased after chronic morphine treatment. Intrathecal administration of sulpiride (8 μg/10 μl) reduced the MOR/D2DR co-localization in the spinal cord (Fig. 6A). In addition, co-immunoprecipitation showed that D2DR interacted with MOR in the mice spinal cord and the MOR/D2DR interactions were enhanced after chronic morphine treatment. Blockade of D2DR with sulpiride (8 μg/10 μl) disrupted the MOR/D2DR interactions in the spinal cord (Fig. 6A and B).

### Chronic morphine treatment increases the expression of D2DR and the MOR/D2DR interactions in primary cultured neuronal cells

**Figure 7:**
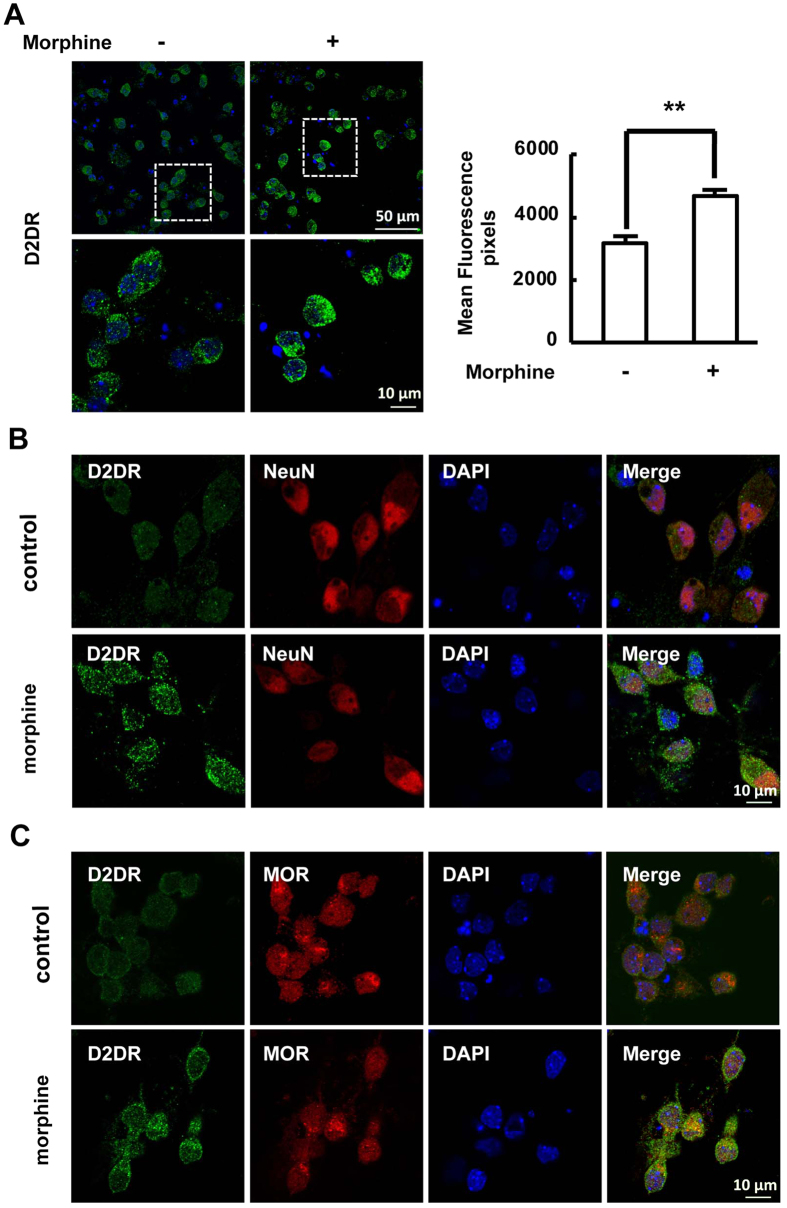
Chronic morphine treatment increases the MOR/D2DR interactions in primary spinal cord dorsal horn neurons. (**A**) Chronic morphine treatment increased the mean fluorescence density of D2DR in primary spinal cord dorsal horn neurons. Quantification of D2DR immunofluorescence was represented as mean fluorescence pixels in the superficial dorsal horns (n = 4, *P < 0.05, **P < 0.01, compared with morphine group). (**B**) Confocal images and immunofluorescence analysis data showed that D2DR (green) was co-localized with NeuN (red) and this co-expression was increased after chronic morphine treatment in primary spinal cord dorsal horn neurons (n = 4). (**C**) Confocal images and immunofluorescence analysis data showed that D2DR (green) was co-localized with MOR (red), and this co-expression was increased after chronic morphine treatment (n = 4).

Primary neuronal cells were cultured to further confirm the effect of chronic morphine administration on the expression of D2DR and interactions of MOR/D2DR *in vitro*. The cells were stained by incubation with primary antibody against the neuron-specific marker NeuN, indicating that we have cultured the appropriate cells. Immunofluorescence staining was used to detect the expression and distribution of D2DR. Consistent with the *in vivo* results, chronic morphine treatment increased mean fluorescence density of D2DR-positive cells and the co-localization of D2DR with MOR or NeuN were also increased after chronic morphine treatment (Fig. 7A and B).

## Discussion

In the present study, we have demonstrated for the first time that chronic morphine administration significantly increase neuronal D2DR protein level in mice spinal dorsal horn. Blockade of D2DR with sulpiride significantly attenuates the development of morphine tolerance or even partly reverses the established morphine antinociception tolerance. These observations strongly support that spinal D2DR play a key role in the development of chronic morphine tolerance.

Dopamine receptors which belong to G protein-coupled receptors have been divided into two sub-families, D1-like and D2-like, on the basis of their pharmacological and biochemical properties. D1-like receptors (including dopamine D1 and D5 receptors) are involved in activating cAMP, while D2-like receptors (including dopamine D2, D3 and D4 receptors) are involved in blocking cAMP^28^. Recently, D2DR was widely accepted to associate with analgesia and morphine antinociception^29^. Anita Verrna and colleagues^30^ reported that D2DR agonist bromocriptine (i.p.) can enhance the ability of MK-801, a non-competitive NMDA receptor antagonist, to attenuate the development of morphine tolerance. Others recommended that both D2DR agonist quinpirole (i.p.)^20^ and D2DR antagonist sulpiride (i.p.)^20^, eticloride (i.p.)^31^ decreased the development of tolerance to antinociception induced by morphine. Therefore, the effect and mechanism of D2DR on morphine tolerance remain unclear.

Our study demonstrated that D2DR mainly expressed in neurons of mice spinal cord and increased significantly after chronic morphine treatment. Blockade of D2DR with sulpiride obviously prevented morphine tolerance and partly reversed the established tolerance induced by chronic morphine exposure. Since sulpiride can antagonize both dopamine D2 and D3 receptor, the specific D2 receptor antagonist L-741,623 and D3 receptor antagonist SB 277011 were used here to determine whether sulpiride attenuated morphine tolerance through antagonizing dopamine D2 receptor. It turned out that L-741,623 could significantly alleviate morphine tolerance, while D3 receptor antagonist SB 277011 did not affect the development of morphine tolerance.

The dopamine D3 receptor has been reported to be related with morphine antinociception and morphine tolerance with D3KO mice^32^,^33^. It is reported that D3KO mice display hyperactivity, increased locomotor activity and hypertension^34^,^35^, and D3KO spinal neurons do not compensate for the loss of function of the D3 receptor with changes in the other DA receptor subtypes^36^. Kori L. Brewer and colleagues reported that the D3KO mice show decreased paw withdrawal latencies and a single morphine administration (2 mg/kg, i.p.) cannot increase withdrawal latencies which may be called morphine-resistant state^32^. However, Tao Li *et al*. indicated that D3KO mice show pronounced hypoalgesia and display enhanced morphine (3 mg/kg and 5 mg/kg but not 1 mg/kg, i.p.) induced analgesia compared with WT mice in tail-flick nociception models^33^, and D3KO mice can develop lower morphine-induced tolerance^33^. These results are controversial and we hypothesized that the different responses to morphine may as a result of the different doses of morphine used in these reports. On the other hand, some other reports suggested that intraperitoneal administration of D3 receptor antagonist can attenuate morphine tolerance^37^. Since morphine or D3 receptor antagonist were systemic (i.p.) administered in all of the above reports, the effect of spinal D3 receptor on morphine tolerance was still not clear. Herein, our study indicated that intrathecal administration of D3 receptor antagonist SB 277011 could not attenuate morphine tolerance, which suggested that the spinal D3 receptor may not contribute to morphine tolerance.

As opioid tolerance and pathological pain share the common cellular mechanisms^38^, chronic morphine treatment can lead to the spinal neuronal sensitization and neuroinflammation. Compelling evidences have accumulated that chronic morphine treatment increases the levels of phosphorylation of PKC, NR1 in spinal cord^5^,^6^. Inhibition of spinal D2DR reduced the expression of these up-regulated proteins. These findings suggested that D2DR may govern dorsal horn neuronal adaptation during chronic morphine exposure. It should be noted that PKC can be activated by D2DR^39^. PKC is widely accepted as a downstream of NMDA receptor in morphine tolerance, however, the activated PKC is also reported can activate NMDA receptor to further promote the central sensitization^40^.

The MAPK family which includes extracellular signal regulated protein kinase 1/2 (ERK1/2), p38 and c-Jun N-terminal kinase (JNK) is reported to be involved in morphine tolerance^7^,^8^,^9^,^41^,^42^. Our results indicated that blockade of D2DR effectively decreased the up-regulated levels of phosphorylated MAPKs in the spinal cord after chronic morphine treatment.

The expression of ERK primarily distributes in neurons, while JNK mainly localizes in astrocytes, and p38 mainly distributes in microglia^43^. Blockade of D2DR here could suppress the up-regulated p-JNK and p-p38. Numerous investigators have demonstrated that chronic morphine treatment can directly or indirectly activate glias^44^,^45^,^46^,^47^, the activated astrocytes and microglia release pro-inflammatory cytokines such as TNF-α, IL-1β, IL-6, and chemokines in spinal cord. These cytokines can bind to receptors on neurons to further sensitize the NMDA receptor, thereby promoting the development of morphine tolerance^48^. Herein, blockade of D2DR suppressed the activation of astrocytes and microglia and decreased the release of pro-inflammatory factors TNF-α and IL-1β after chronic morphine treatment. However, the D2DR was not expressed in astrocytes and microglia, suggesting that the glias were indirectly suppressed by D2DR antagonist probably through the inhibition of neuronal sensitization.

Accumulating evidences have suggested that activation of JNK and p38 MAPK in spinal dorsal horn contributes to the induction and maintenance of chronic pain^43^. We proposed that the activation of JNK and p38 MAPK was also involved in the maintenance of morphine tolerance^38^. In addition, the PKC is also a key player in the maintenance of morphine tolerance through desensitizing the MOR^3^,^49^. Blockade of D2DR in this report could decrease the up-regulated p-PKC, p-JNK and p-p38, and reverse the established tolerance in a dose dependent manner.

Interestingly, the immunofluorescence analysis showed that D2DR and MOR were co-localized both *in vivo* and i*n vitro* in this study. The co-immunoprecipitated results showed that D2DR interacted with MOR and these interactions were increased after chronic morphine treatment. Blockade of D2DR in the spinal cord disrupted the interactions of MOR/D2DR and attenuated morphine tolerance suggesting that the increased MOR/D2DR interactions may play a critical role in chronic morphine tolerance.

Opioid receptors may heteromerize with a wide range of GPCRs including DOR^50^, adrenergic^51^, cannabinoid^52^ metabotropic glutamate^53^ and sensory neuron specific receptors^54^ which may be involved in opioid analgesia, tolerance and reward. Jason R. Juhasz and coworkers visualized directly that MOR form heteromeric oligomer with the dopamine D1 receptor in living cells^55^. We speculated that the interactions of D2DR with MOR in spinal neurons lead to the desensitization of MOR and regulate MOR activity.

As previously mentioned, PKC can phosphorylate MOR to desensitize it after chronic morphine treatment. We hypothesized that the MOR/D2DR complexes mediated recruitment of PKC to promote the development of morphine tolerance and may switch in MOR coupling from Gi to Gz to promote morphine tolerance similar to MOR/DOR heterodimer^56^.

In summary, the present study suggests that chronic morphine treatment increases D2DR protein expression and D2DR can interact with MOR in mice spinal cord, which may desensitize the MOR to promote the morphine tolerance. Blockade of D2DR in spinal cord can disrupt the interactions between MOR and D2DR to attenuate morphine tolerance. These findings highlight the possibility of a new clinical strategy to prevent morphine antinociceptive tolerance.

## Methods

### Experimental animals

The study was approved by the Animal Experimentation Ethics Committee of China Pharmaceutical University and performed in accordance with the guidelines of the International Association for the Study of Pain. Adult male ICR mice weighing 18–22 g at 8–10 weeks of age (the Experimental Animal Center at Nanjing Medical University, China) were free to the food and water and housed in a 12 h light/dark cycle at 22 °C. The behavior test was blinded.

### Western blot

Tissue extracts were analyzed as described before^11^,^57^. In brief, tissues (spinal cord segments at L4-L6 of ICR mice) were collected and washed with ice-cold 0.01 M phosphate buffered saline (PBS) before being lysed in RIPA. Then whole lysates containing 50 μg proteins were separated on sodium dodecyl sulfate-polyacrylamide gels, and transferred onto polyvinylidenedifluoride membranes (Millipore, Billerica, MA). The membranes were blocked with 5% BSA (Sunshine Biotechnology, Nanjing, China) for 2 h at room temperature and incubated overnight at 4 °C with the primary antibodies GAPDH (1:8000, Sigma-Aldrich, St. Louis, MO, USA); GFAP (1:1000, Millipore, Billerica, MA, USA); IBA-1 (1:1000, Wako Pure Chemical Industries, Osaka, Japan); IL-1β (1:1000, Santa Cruz Biotechnology, CA, USA); p-NR1 (Ser896), p-PKC, p-ERK, p-JNK, TNF-α, p-p38 (Tyr182) (1:1000, Cell Signaling Technology, MA, USA). Then, the membranes were washed with 0.05% TBST and incubated for 2 h at room temperature with the secondary antibodies (1:1000, Cell Signaling Technology, MA, USA). Signals were finally detected using ECL reagents (PerkinElmer, Waltham, MA, USA). Data were analyzed with the Molecular Imager (Gel DocTM XR, 170-8170) and the associated software Quantity One-4.6.5 (Bio-Rad Laboratories).

### Immunofluorescence and Cell immunofluorescence

For immunofluorescence, mice were anesthetized and perfused with 0.01 M PBS followed by 4% paraformaldehyde on day 7. Lumbar spinal cords (L4-L6) were collected and post-fixed in the same 4% paraformaldehyde for one day and then cryo-protected in 30% sucrose for 3–5 days. The embedded blocks were sectioned as 25 μm thick^58^. Tissue sections from each group (4 mice, 8 images in each group) were blocked with 10% normal donkey serum and 0.3% Triton-X-100 (Sigma-Aldrich, St. Louis, MO, USA) for 2 h at room temperature. Then primary antibodies against mouse anti-D2DR (1:50, Santa Cruz Biotechnology, CA, USA), goat anti-IBA1 (1:200, Abcam, Cambridge, MA, USA), rabbit anti-GFAP (1:200, Abcam, Cambridge, MA, USA), or rabbit anti-NeuN (1:200, Abcam, Cambridge, MA, USA), rabbit anti-MOR (1:500, Neuromics, *Edina, Minnesota, USA*) were incubated overnight at 4 °C. Rabbit IgG (1:300; Jackson ImmunoResearch Laboratories, Inc., PA, USA) was used as an isotype control. Then the free-floating sections were washed with PBS, and incubated with the secondary antibody for 2 h. After washing out three times with PBS, the slices were incubated with DAPI (Southern Biotec, Birmingham, Alabama, USA).

For cell immunofluorescence, the cells in confocal dishes from each group (4 confocal dishes in each group) were washed with 0.01 M PBS and fixed in 4% paraformaldehyde at room temperature for 15 min, and then blocked with 10% normal donkey serum in 0.01 M PBS at room temperature for 2 h. Next, the cells were incubated with primary antibodies: rabbit anti-NeuN (1:200, Abcam, Cambridge, MA, USA), mouse anti-D2DR (1:50, Santa Cruz Biotechnology, CA, USA) and rabbit anti-MOR (1:500, Neuromics, *Edina, Minnesota, USA*) overnight at 4 °C. Rabbit IgG (1:300; Jackson ImmunoResearch Laboratories, Inc., PA, USA) was used as an isotype control. Then the cells in confocal dishes were washed with 0.01 M PBS and incubated with the secondary antibody for 2 h. After washing out three times with PBS, the cells were incubated with DAPI (Southern Biotec, Birmingham, Alabama, USA).

Confocal microscopy of dual antibody in both immunofluorescence and cell immunofluorescence were performed with a confocal laser-scanning microscope (Carl Zeiss LSM710, Germany). Images were randomly coded and transferred to a computer for further analysis. To obtain quantitative measurements, 8 images for each group were evaluated and photographed at the same exposure time to generate the raw data. Fluorescence intensities of the different groups were analyzed using Image Pro Plus 6.0 (Media Cybernetics, Silver Spring, MD, USA).

### Primary cultures neurons of spinal cord superficial dorsal horn and drug treatments

Pregnant mice were euthanized by CO_2_ asphyxiation on day 13 of gestation under deep anesthesia. The spinal cords of the embryonic mice were removed aseptically, and digested in 0.15% trypsin (Invitrogen Inc. Carlsbad, CA, USA) at 37 °C for 7 min. The cell suspension was centrifuged at 1000 rpm for 4 min at room temperature. The supernatant was removed with a Pasteur pipette, the cells were resuspended and dissociated by slowly pipetting up-and-down 4–6 times with the smallest fire-polished glass Pasteur pipette. The cell suspension was sedimentated and centrifuged at 1000 rpm for 4 min. Neurobasal plating medium (Gibco, Gaithersburg, MD, USA) containing 10% FBS supplement (Gibco, Gaithersburg, MD, USA), 1% L-glutamine (Gibco, Gaithersburg, MD, USA) were added. 16–18 h later, changed the media to Neurobasal Growth Media containing 10% B27, 1% L-glutamine (Gibco, Gaithersburg, MD, USA) and 1% HEPES. Cells were seeded in polylysine-pretreated confocal dishes with a density of 1.2–1.8 × 10^5^ cells/well^59^. To simulate the morphine tolerance *in vitro*, 10 μM morphine were added to the cells (every other day) for 6 days. In the control group, a same volume of saline as that in the morphine group was added into the culture. Then the expression of D2DR and D2DR/MOR complexes are tested by cell immunofluorescence.

### Behavioural assessment of antinociceptive effects

The antinociception of mice were test by tail-flick test as previously described^60^. A cut-off time of 10 s was set to avoid tissue damage. Data were calculated as percentage of maximal possible effect (% MPE), which was calculated by the following formula: 100% × [(Drug response time − Basal response time)/(10s − Basal response time)] = % MPE. The experimenters were blinded to the treatment.

### Chronic morphine tolerance tests and drug delivery

Repetitive morphine injection (10 μg/10 μl, i.t., Shenyang First Pharmaceutical Factory, Shenyang, China) was given daily for 7 days to lead to the morphine tolerance. The analgesic effect was measured (52 °C water bath) 30 min after each injection. Dopamine receptor antagonist sulpiride (1, 4 or 8 μg/10 μl, i.t., Tocris, Bristol, UK) were dissolved in DMSO and diluted in saline (final concentration of DMSO for i.t. administration was 1%). L-741,626 (1, 4 or 8 μg/10 μl, i.t., Sigma-Aldrich, St. Louis, MO, USA) were dissolved in 25% DMSO, and SB 277011A (2.5, 5 and 10 μg/10 μl, i.t., Abcam, Cambridge, MA, USA) were dissolved in saline. Sulpiride, L-741,626 and SB 277011A were administered intrathecally daily at 15 min before the morphine challenge at 10 μg/10 μl. Groups were divided according to drug treatment: control, morphine, 1 μg sulpiride/L-741,626 or 2.5 μg SB 277011A plus morphine, 4 μg sulpiride/L-741,626 or 5 μg SB 277011A plus morphine, 8 μg sulpiride/L-741,626 or 10 μg SB 277011A plus morphine, 8 μg sulpiride/L-741,626 or 10 μg SB 277011A and 1% DMSO groups/25% DMSO or saline plus morphine (12 mice in each group). In the control group, a same volume of saline as that in the morphine group was given to the mice.

The drugs were injected intrathecally (each in 10 μl) respectively by means of lumbar puncture at the intervertebral space of L4-5 or L5-6 for multiple injections using a stainless steel needle (30 gauge) attached to a 25 μl Hamilton syringe^11^.

### Co-immunoprecipitation

To verify the interactions of D2DR and MOR, 10 μg mouse anti-D2DR (Santa Cruz Biotechnology, CA, USA) or goat anti-MOR (Santa Cruz Biotechnology, CA, USA) was added to the NHS magnetic beads (Enriching biotechnology, Nanjing, Jiangsu, China), and incubated with rotation for 4 h at 4 °C in 500 μl coupling buffer. Then the beads-Ab complex was incubated in 500 μl blocking buffer for 1 h at 4 °C. Tissues (spinal cord segments at L4-L6 of ICR mice) were lysed in ice-cold RIPA buffer (150 mM NaCl, 30 mM HEPES, 10 mM NaF, 1% Triton, 0.01% SDS), the suspended lysate was immunoprecipitated with the beads-Ab complex overnight at 4 °C. Immunoprecipitates were collected and resuspended in RIPA buffer without SDS, washed at least three times, and then 100 μl elution buffer was added and incubated with rotation for 5 min at room temperature to dissociate the complex. The supernatant was transfered and incubated in SDS sample buffer for 10 min at 100 °C. Then separated it on sodium dodecyl sulfate-polyacrylamide gels, and transferred onto polyvinylidenedifluoride membranes (Millipore, Billerica, MA).

### Statistical analysis

SPSS Rel 15 was used to conduct all the statistical analyses (SPSS Inc., Chicago, IL, USA). Alteration of expression of the proteins detected and the behavioral responses to morphine withdrawal were tested with one-way analysis of variance (ANOVA) and the differences in latency over time among groups were tested with two-way ANOVA, followed by Bonferroni’s post-hoc tests. Results are expressed as mean ± standard error from three independent experiments in triplicate. Results described as significant are based on a criterion of *P* < 0.05 was considered significant.

### Ethics approval

The study was approved by the Animal Experimentation Ethics Committee of China Pharmaceutical University and performed in accordance with the guidelines of the International Association for the Study of Pain.

## Additional Information

**How to cite this article**: Dai, W.-L. *et al*. Blockade of neuronal dopamine D2 receptor attenuates morphine tolerance in mice spinal cord. *Sci. Rep.* **6**, 38746; doi: 10.1038/srep38746 (2016).

**Publisher's note:** Springer Nature remains neutral with regard to jurisdictional claims in published maps and institutional affiliations.
